# Synthesis of Cellular Silica Using Microbubbles as Templates

**DOI:** 10.3390/nano12162794

**Published:** 2022-08-15

**Authors:** Zirui Zhao, Jiamei Liu, Xifeng Xi, Yulong Wu, Junshe Zhang

**Affiliations:** 1 School of Chemical Engineering and Technology, Xi’an Jiaotong University, Xi’an 710049, China; 2 Instrumental Analysis Center, Xi’an Jiaotong University, Xi’an 710049, China; 3 Institute of Nuclear and New Energy Technology, Tsinghua University, Beijing 100084, China

**Keywords:** cellular silica, microbubbles, silicon tetrafluoride, large pore, surface tension

## Abstract

In this work, cellular silica was synthesized by using microbubbles as templates, which contain a mixture of argon and silicon tetrafluoride (SiF_4_). The latter is generated from decomposition of hexafluorosilicic acid (H_2_SiF_6_) at ambient conditions. The specific surface area of cellular silica can be as high as 130 m^2^/g, the size of the cavity is hundreds-of-nanometers, and the thickness of the cavity wall is around 30 nm. The cavity size, apparent packing density, and porosity of cellular silica strongly depend on the nature of the aqueous solutions; the cavity size appears to be negatively proportional to the surface tension, but thickness of cavity walls seems to be weakly affected by the aqueous properties. An attempt was made to introduce aluminum atoms in situ in the second-coordination sphere of Si atoms and/or load aluminum into the silica structure. Cellular silica with large pores facilitate the transfer of large molecules, including polymers and enzymes; thus, it could find applications in (bio)catalysis, sorption, controlled release and separations.

## 1. Introduction

Among porous solids, silica-based materials with tailored pore dimensions and high surface area, high chemical and thermal stabilities, and good compatibilities with other materials, have attracted a great deal of attention because of their possible uses as adsorbents, catalyst supports, dielectric materials, optical materials, polymer fillers, templates, and therapeutic agents [1]. Based on the geometry and topology of pores, porous silica can be classified into regular and disordered porous materials. The former has regular and ordered porous networks while the latter has random porous networks. Regular porous silica with amorphous pore walls, such as MCM-41 and SBA-15, have gained wide popularity over the past two decades. MCM-41 and SBA-15 are mesoporous silica materials composed of periodic arrangements of man-sized hexagonal pores. The pore diameters of MCM-41 and SBA-15 are 2.5–6 and 6.6–30 nm, respectively [2]. The uniform and tunable pore size, good biocompatibility, easy functionalization of the surface, and the gating mechanism of the pore opening make mesoporous silica nanoparticles (MSNs) excellent carriers for loading a variety of cargo, ranging from drugs to biomacromolecules, including enzymes, antibodies and nucleic acid by electrostatic, hydrophobic, hydrogen-bonding, or other non-covalent interactions between the cargo molecules and the silica surface [2,3,4,5,6,7,8,9].

The aforementioned MSNs have little morphological flexibility because of rigid pore walls; thus, the effective binding and release generally involves precise molecular design. Although the pore dimension of MSNs can be easily tailored to accommodate small guest molecules, it cannot always be adjusted to load large molecules. Moreover, particles with large pore sizes may give rise to unexpected release in the absence of surface modifications. In these regards, a porous substrate that has easily accessible spaces of appropriate dimensions and morphological flexibility might be more suitable for encapsulation of atoms, molecules, or nanostructures. It was demonstrated that a porous silica comprising nanosheets of 10 nm in thickness is capable of encapsulating various molecular species without any surface modification [10].

Porous silica comprising nanosheets can be synthesized by magnetron sputtering deposition, soft-templating approach [11,12], and selective etching [13]. These methods always involve multiple steps and/or complicated procedures; thus, production cost is relatively high. Therefore, it remains necessary to explore other processes that can be used for industrial scale-up production of porous silica. In this context, we investigated the hydrolysis of silicon tetrafluoride (SiF_4_) that is generated from decomposition of hexafluorosilicic acid (FSA, H_2_SiF_6_), a by-product of phosphoric acid manufacturing process. Currently, FSA is used for drinking water fluorination, manufacture of aluminum fluoride, cryolite, and other fluorosilicates. However, there are more than two million tons of FSA (reported as 100% FSA) that need to be disposed annually [14]. As a result, the disposal of liquid waste is challenging manufacturers to both comply with environmental protection laws and reduce neutralization or disposal costs. Production of porous silica, a high value-added commodity, from FAS opens new market outlets for phosphoric acid producers.

Generally, SiO_2_ synthesized from hexafluorosilicic acid involves two steps: decomposition (H_2_SiF_6_ → SiF_4_ + 2HF) and hydrolysis (3SiF_4_ + 2H_2_O → 2H_2_SiF_6_ + SiO_2_). The overall reaction is H_2_SiF_6_ + 2H_2_O → 6HF + SiO_2_. The above reactions suggest that the single-pass conversion is 33.33%; thus, recycling is required to totally convert hexafluorosilicic acid into silica. Herein, we predominantly focus on the nature of silica, which was poorly investigated in previous studies, and emphasis was placed on the effect of slats on its physical properties. The cellular silica prepared by this method has a large pore volume, and its cavity size could be easily tuned. This three-dimensional mesoporous/macroporous material has a wide range of applications in the food and pharmaceutical industries. In many current studies, porous supports have been widely used to immobilize enzymes due to their high porosity and high surface area. Because enzymes are large molecules, they can only be immobilized on the outer surface of the porous support if its pore size is small. Mesoporous materials with larger pore volume offer the possibility to support larger volumes of target materials. On the other hand, larger pores facilitate the diffusion of reactive molecules [15,16,17]. As with other porous silica, cellular silica can also be used as a support for catalytically active species [18,19,20]. Usually, either one-pot synthesis or post-loading are used to endow silica with acidic sites or metal active centers [21,22,23,24]. To achieve that, we tried to directly introduce Al into the framework of silica or support Al in the silica structure. The obtained cellular silica was characterized by various techniques, including N_2_ physical adsorption, scanning electron microscope, and nuclear magnetic resonance. The specific surface area of cellular silica can be as high as 130 m^2^/g, the cavity size hundreds-of-nanometers, and the thickness the of cavity wall around 30 nm. It seems that the cavity size is negatively proportional to the surface tension, but thickness of cavity walls is weakly affected by the aqueous properties. These findings may provide an approach to produce high value-added porous silica from the by-products of the phosphoric acid manufacturing process.

## 2. Experimental Section

### 2.1. Materials

Fluorosilicic acid (H_2_SiF_6_, 33–40%), sulfuric acid (H_2_SO_4_, 95%), and aluminum nitrate nonahydrate (Al(NO_3_)_3_·9H_2_O) were purchased from Aladdin Biochemical Technology (Shanghai, China). Deionized water was provided by Tianyi Inc (Xi’an, China). Argon (Ar, 99.999%) was supplied by Tenglong Inc (Xi’an, China).

### 2.2. Synthesis

Typically, 20 mL fluorosilicic acid was continuously injected into a 150 mL Teflon batch reactor by a syringe pump, where 100 mL sulfuric acid was placed, and the volumetric flowrate of feeding was 350 μL min^−1^. The decomposition was carried out at ambient conditions. The gaseous products were carried to another Teflon condenser (150 mL) by argon (10 STP mL min^−1^) which contained 100 mL sulfuric acid. The condenser was placed in a thermostat with the temperature set to −30 °C. After passing through the concentrated sulfuric acid, HF was removed from the gaseous stream. After that, the gas mixture was sparged into an aqueous solution through a plastic nozzle (Figure S1). The hydrolysis of SiF_4_ was performed at ambient conditions. The samples were prepared in pure water and 0.1 M Al(NO_3_)_3_ solution, respectively. The surface tensions of the 0.06 M H_2_SiF_6_ solution without and with 0.1 M Al(NO_3_)_3_ are 67.24 and 91.43 mN/m (Figure S2), respectively. After the hydrolysis, the solid was separated from the liquid phase by passing the suspension through a filtering media, followed by washing the cake with D.I. water. The wet cake was then dried in an oven at 120 °C overnight. The obtained power was stored in a glass vial at ambient condition. 

### 2.3. Surface Tension

The surface tension is measured by the pendant drop method on KRUSS-DSA100. In the pendant drop method, the surface or interfacial tension is calculated from the shadow image of a pendant drop using the shape analysis [25]. The aqueous solution was introduced slowly into the air through a needle tube placed vertically. This aqueous solution formed a pendant drop at the tip of the needle. The images of the droplet were captured with the digital camera and digitized by the image analysis system; an algorithm was then used to analyze the digital image to determine the surface tension. The surface tension is related to the drop shape by the equation *γ* = Δ*ρgD*_0_^2^/*H*, where Δ*ρ* is the density difference between the air and drop (g/cm^3^), *g* represents the gravity acceleration (cm/s^2^), *D*_0_ is the large diameter of the drop, and H stands for the shape factor of the drop.

### 2.4. Characterization

The microstructure of silica samples was examined by a TESCAN MALA3 LMH (Warrendale, PA, USA) scanning electron microscope (SEM) that was operated at an accelerating voltage of 5–15 kV. The sample was ground and then dusted onto an adhesive conductive carbon belt attached to the sample holder, and gold was deposited on the samples.

The X-ray diffraction (XRD) patterns of silica samples were collected on a D/Max-R diffractometer (Shimadzu LabX XRD 6100 laboratory, Tokyo, Japan) equipped with a Cu Kα radiation source (λ = 0.15406 nm). The working voltage and current of the diffractometer are 40 kV and 30 mA, respectively. The fluffy powders were firmly pressed into the recess of a sample holder, followed by flushing the surface of the powder bed with the sample holder edge. The sample was scanned at a 2θ range of 10−90° with a rate of 5 °/min.

N_2_-physisorption on silica samples at −196 °C was carried out on a volumetric adsorption analyzer (Micromeritics ASAP 2460, Atlanta, GA, USA). Before the adsorption, the sample was degassed at 300 °C for 3 h under vacuum. The specific surface area was calculated by using the Brunauer–Emmett–Teller method at a relative pressure range of 3 × 10^−6^ to 0.2 and the pore size distribution was estimated from the desorption branch of isotherms.

The elemental composition of silica samples was estimated from X-ray fluorescence (XRF) spectroscopy, which was collected on a wavelength-dispersive XRF spectrometer (Bruker S8 TIGER, Karlsruhe, Germany) that was equipped a 4 kW solid-state generator and water-cooled X-ray tube, a 170 mA maximum excitation current, and a 60 kV maximum acceleration voltage. Spectrum recording and evaluation were carried out with the pre-calibrated/standardless software Quant-Express. Before measurements, the as-prepared sample was placed into a die which was then pressed.

The solid-state ^27^Al and ^29^Si magic-angle spinning (MAS) nuclear magnetic resonance (NMR) measurements were carried out on a JEOL JNM-ECZ600R (Tokyo, Japan) spectrometer with the Larmor frequencies of 156 and 119 MHz, respectively. The spectrometer was equipped with a 3.2 mm probe. All spectra were acquired using 12 kHz rotating speed via the small-flip angle method with a single pulse (π/6) length of 0.5 μs plus a recycle delay of 5 s. The collected NMR data were processed using Delta software.

## 3. Results and Discussions

**Figure 1:**
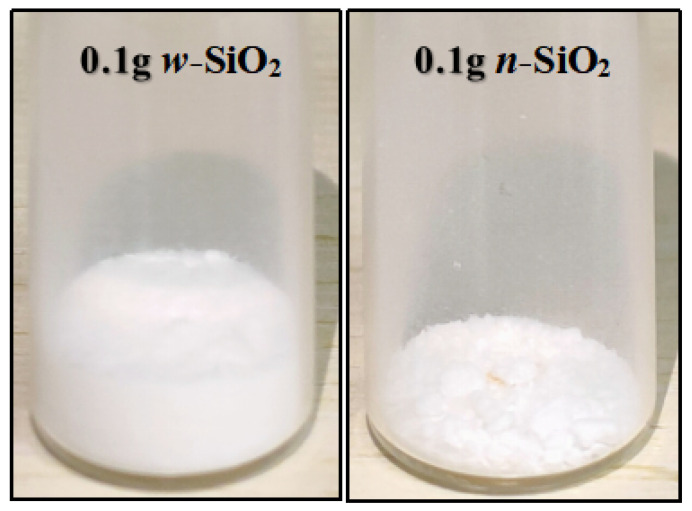
Images of *w*-SiO_2_ and *n*-SiO_2_ samples.

Silica samples synthesized in pure water and 0.1 M Al(NO_3_)_3_ solution are denoted as *w*-SiO_2_ and *n*-SiO_2_, respectively. Figure 1 illustrates the images of *w*-SiO_2_ and *n*-SiO_2_. Obviously, the former is fluffier than the latter. It seems that the bulk density of *n*-SiO_2_ particles is at least two times as high as that of *w*-SiO_2_ ones. 

**Figure 2:**
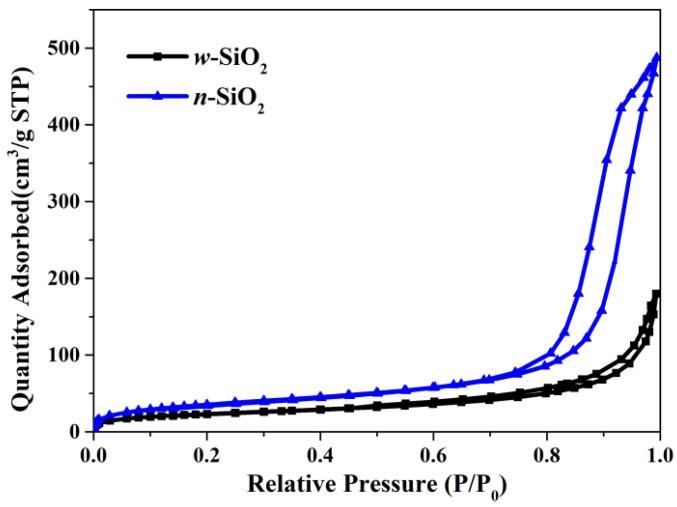
Adsorption and desorption isotherms of nitrogen on *w*-SiO_2_ and *n*-SiO_2_ samples.

**Figure 3:**
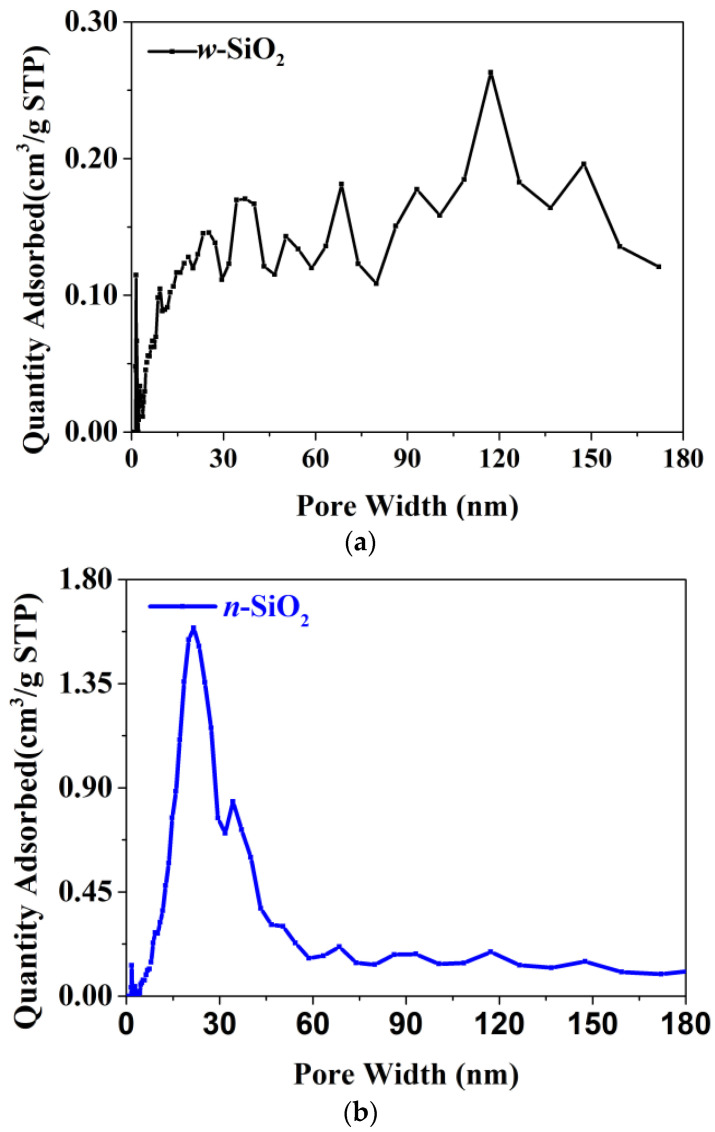
The pore size distribution of *w*-SiO_2_ (**a**) and *n*-SiO_2_ (**b**) samples.

Another interesting finding is that the N_2_-BET surface area of *w*-SiO_2_ is smaller than that of *n*-SiO_2_. Specifically, the former is 83 m^2^/g, while the latter is 130 m^2^/g. The N_2_ adsorption isotherms are presented in Figure 2. According to the IUPAC classification, both samples give a Type IV(a) isotherm with an H1 hysteresis loop whose low closure point is located at a relative pressure above 0.45. The loop given by *n*-SiO_2_ is much larger the one given by *w*-SiO_2_, suggesting the pore size of these two samples are very different. To shed more light on the pore size, BJH analysis using the desorption branch was carried out and the pore size distribution (PSD) in the differential form is presented in Figure 3. For *w*-SiO_2_, the distribution is very broad (from ~5 to ~170 nm) and there are multiple small peaks on the PSD curve. However, *n*-SiO_2_ displays a bimodal PSD, a large peak centered at ~20 nm and very tiny peak with a maximum ca. 30 nm. 

**Figure 4:**
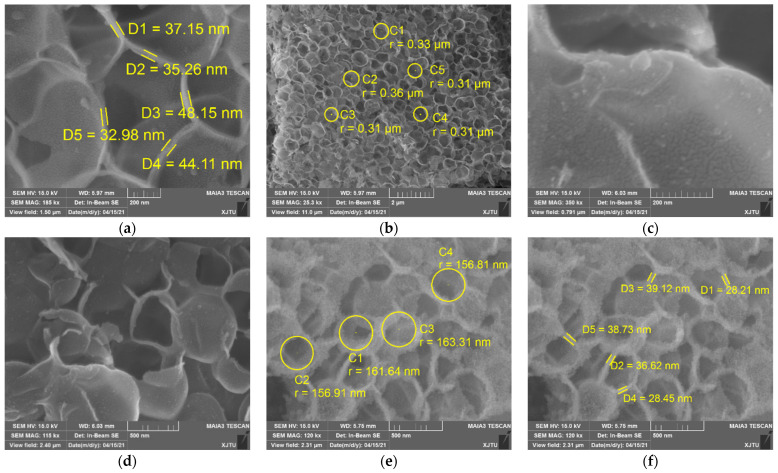
SEM images *w*-SiO_2_ (**a**–**d**) and *n*-SiO_2_ (**e**,**f**) samples.

To obtain more information about the morphology of silica, the samples were characterized by scanning electron microscopy (SEM). As can be seen, silica synthesized from SiF_4_ hydrolysis has cellular structures, comprising of connected irregular curved sheets without clear periodic patterns (Figure 4). A close examination of the SEM images reveals that the wall of a cavity is composed of nano-sized silica particles. This structure is similar to the surface of a lotus leaf, covered with many tiny papillae [26]. Besides open cavities, there exist a small number of closed cavities. Cavities are closed or open, which depends on whether both edges and faces are solid or only the cavity edges are solid. Open cavity cellular materials are permeable to the flow of fluids, which is usually a prerequisite in biomedical applications [27]. On the other hand, closed cavity ones are preferred when insulating (thermal, acoustic) properties are required. For cellular silica, a substantial part of its bulk volume is occupied by the void volume (filled with air). Aluminum nitrate has a little effect on the thickness of cavity walls, ranging from 30 to 50 nm and from 20 to 40 nm for *w*-SiO_2_ and *n*-SiO_2_, respectively. However, the size of cavities is strongly dependent on whether aluminum nitrate is present or not. The average diameters of *w*-SiO_2_ and *n*-SiO_2_ are about 300 and 150 nm, respectively. This may be because the cavity size is determined by the size of bubbles, which is dependent on the surface tension of aqueous solutions. The higher the surface tension, the smaller is the size. This can be inferred from the theoretical calculation. The additional pressure generated by the curved liquid surface of the bubbles in the liquid can be calculated by the Laplace pressure equation (Δ*p* = 2γ/*r*, where Δ*p* is the pressure difference between the inside of a bubble and the outside, γ is the surface tension of the gas-liquid interface, *r* is the radius of the bubble). When the pressure difference is fixed, the radius of the bubbles is negatively proportional to the surface tension. According to SEM observations, a growth mechanism of cellular silica in the aqueous phase is proposed. 

**Figure 5:**
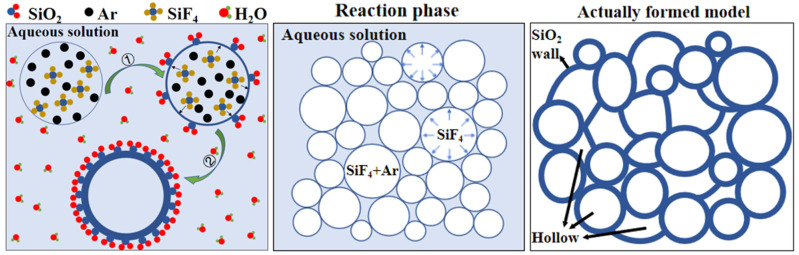
Growth mechanism of cellular silica in aqueous solution.

When the gas mixture of SiF_4_ and argon is sparged into the aqueous phase through a nozzle, gas bubbles form. According to the stoichiometry, the gas phase composition of the bubbles is about 80–85 vol% argon and 15–20 vol% silicon tetrafluoride. Based on the diameter and the thickness of the individual cavities, we estimated the size of gas bubbles before the hydrolysis starts. The following assumptions were made when calculating the initial bubble size: (1) the temperature and pressure of the gas mixture is 298 K and 1 atm, (2) the gas mixture follows the ideal gas behavior, (3) water vapor is ignorable, and (4) the density of cavity walls is 2.2 g/cm^3^. As soon as the gas bubbles make contact with water, SiF_4_ hydrolysis starts, followed by condensation which yields silica particles at the nanoscale (Figure 5). 

**Figure 6:**
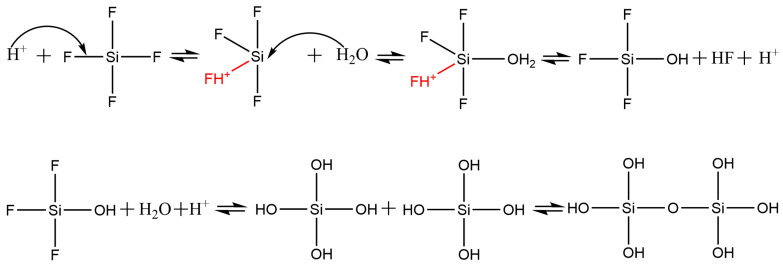
Reaction mechanism of silicon tetrafluoride hydrolysis.

The mechanism of SiF_4_ hydrolysis is illustrated in Figure 6. The proton activates the F group of SiF_4_ molecules, which causes the electron cloud of SiF_4_ molecules to shift to the protonated side. As a result, Si atoms that have 3D empty orbitals accept the lone pair of electrons of water molecules. Subsequently, hydrolysis proceeds. The orthosilicic acid and other intermediates produced by the hydrolysis undergo a series of polycondensation reactions, and finally Si–O–Si bonds forms. These nanoscale particles adjoin each other, forming the walls of cavities. As the hydrolysis proceeds, the thickness of walls increases but the size of cavities changes slightly. We surmise that the hydrolysis is extremely fast compared with the movement of cavities in the aqueous phase, and it stops when the diffusion of gases outside the cavities is terminated. As the cavities grow and aggregate, most cavities lose their spherical shape and turn into irregular ones. In the aggregates, one cavity shares the wall with others (Figure 5). Upon combination of SEM and BET measurements, we conclude that the cellular silica belongs to mesoporous materials and thus it may find broad applications in sewage treatment, catalysis, separation and tissue engineering.

**Figure 7:**
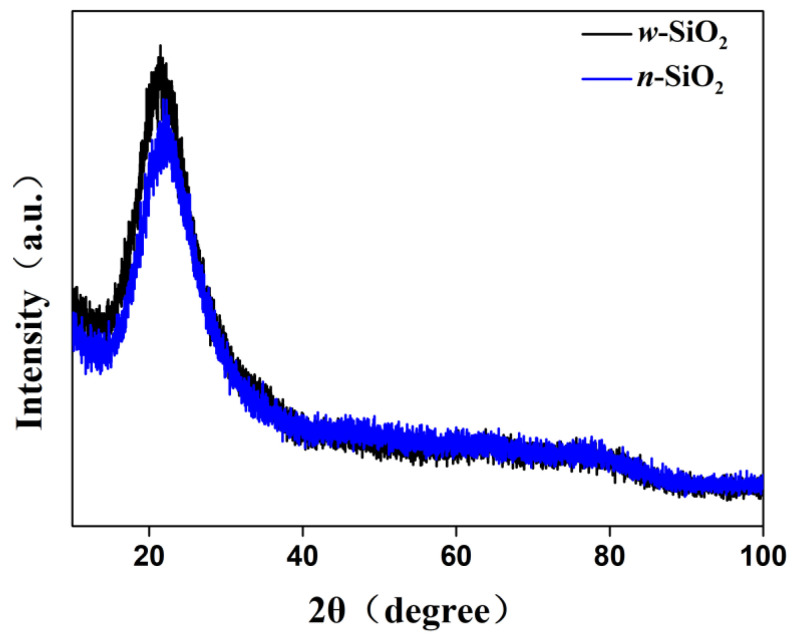
XRD patterns of *n*-SiO_2_ and *w*-SiO_2_ samples.

Besides the morphology and surface properties, the structure and composition of silica were also investigated. Figure 7 illustrates the XRD patterns of *w*-SiO_2_ and *n*-SiO_2_. For both samples, there is only one broad diffraction peak at a 2θ range of 18 to 26°, suggesting that a silica synthesized from SiF_4_ hydrolysis is amorphous. However, the peak of *n*-SiO_2_ slightly shifts to higher diffraction angle compared with that of *w*-SiO_2_. The shift cannot be attributed the substitution of Si cations with Al cations in silica. This is because the radius of aluminum ions is larger than that of silicon ions, the substitution causes the structure to expand, resulting in a shift to lower diffraction angle. A possible reason is that a small fraction of Si cations in the surface layer are substituted with Al cations, which may cause a change in the position of atoms or the length of chemical bonds within silica. The elemental composition of *n*-SiO_2_ was determined by X-ray fluorescence (XRF) spectroscopy technique and energy-dispersive X-ray spectroscopy (EDS). The atomic ratios of Al to Si obtained from bulk XRF and SEM-EDS measurements are 1:13.8 and 1:6.4, respectively (Figure S3 and Table S1). The difference could be due to how SEM-DES analysis determines the atomic ratio of a selected area, whereas XRF analysis provides the elemental composition of the whole area. Although aluminum atoms are detected in *n*-SiO_2_, whether it occurs as an individual phase of aluminum oxide or other species remains unknown. To shed light on that, we used solid-state nuclear magnetic resonance (NMR) spectroscopy to elucidate the bonding arrangements of silicon and aluminum atoms in *n*-SiO_2_. 

**Figure 8:**
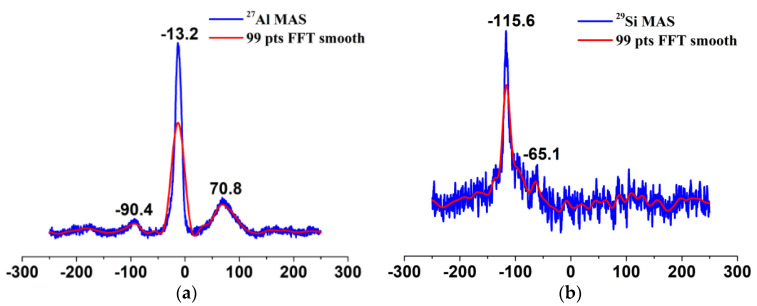
^27^Al MAS−NMR spectrum (**a**) and ^29^Si MAS spectrum (**b**) of *n*−SiO_2_ sample: the blue lines are the original data, and the red ones are the fitted data.

The basic structural units of silicates and aluminosilicates are TO4 tetrahedra with silicon atoms at the center. In the second coordination sphere of these silicon atoms, aluminum atoms can be incorporated into the framework. Depending on the number of aluminum atoms incorporated, the tetrahedrally coordinated silicon atoms (Q^4^) can have five different environments denoted as Si(*n*Al) with *n* = 0,1,2,3, and 4. Each type of Si(*n*Al) species has a characteristic chemical shift [28,29]. On the other hand, hydroxy groups can bound to silicon atoms at the outer surface or at the internal defects, yielding Q^2^(Si(OSi)_2_(OH)_2_) and Q^3^(Si(OSi)_3_OH) units [28]. These sites can be distinguished based on the ^29^Si MAS chemical shifts. As illustrated in Figure 8b, the ^29^Si MAS spectrum consists of a broad signal that is composed of three unresolved peaks with isotropic chemical shifts of ca. −114, −115 and −116 ppm, arising from the tetrahedrally coordinated silicon atoms (Q^4^, Si(OSi)_4_). Therefore, silanol groups may be absent in *n*-SiO_2_ because one silanol group always gives rise to an upfield chemical shift of ~10 ppm for silicates and aluminosilicates (The chemical shifts of ^29^Si for Q^4^, Q^3^, and Q^2^ species are ~−110 ppm, ~−100 ppm and ~−90 ppm, respectively [30].). At the same time, adding one tetrahedrally coordinated aluminum atom in the structure of Si(*n*Al) species (Si[(OAl)_n_(OSi)_4−n_]) leads to a chemical shift of ca. 5 ppm to positive values [29], so for the chemical shift of ca. −114, −115 and −116 ppm, we can think that in *n*-SiO_2_, the existence state of Si is dominated by Q^4^ (0Al). For porous materials, however, the line broadening due to dipolar interactions and chemical shift distribution may be so large that the signals of Si(*n*Al) species with *n* ≥ 1 cannot be resolved. For *n*-SiO_2_, we surmise the Si(*n*Al) with *n* ≥ 1 may be absent. Figure 8a shows the ^27^Al MAS NMR spectrum of *n*-SiO_2_. The ^27^Al spectrum contains two broad resonances; one is centered at 70.8 ppm and the other at −13.2 ppm, which are assigned to four-coordinated (or tetrahedrally coordinated) and six-coordinated (or octahedrally coordinated) aluminum species, respectively [31,32]. The intensity ratio of AlO4 to AlO6 is 1:2.05. As mentioned before, we suspect that substituting aluminum atoms for silicon atoms in Q^4^ species is impossible. Thus, aluminum species may occur as an individual phase in *n*-SiO_2_. Aluminum hydroxides (Al(OH)_3_) and oxyhydroxides (AlOOH) exclusively contain hexa-coordinated aluminum ions, leading to an Al^tet^/Al^hex^ ratio equaling zero [31,32]. In *n*-SiO_2_, aluminum species most likely exist in the form of alumina, which are either amorphous or with disordered structures. Generally, the bridging hydroxyl groups (Si–OH–Al) corresponding to framework tetracoordinate aluminum constitute Brønsted acid sites, while the non-framework aluminum species constitute Lewis acid sites. Therefore, whether Al atoms incorporate into the framework or not, it can endow the material with acid sites. However, unequivocal identification of alumina phases requires further studies.

Compared with other methods to synthesize silica materials, the main advantages of this approach include cheap silicon sources, mild operating conditions, no complicated post-processing steps, and little difficulty in scaling-up (Table 1). Moreover, this method can easily regulate the size of the cellular silica by adjusting the surface tension of the aqueous solutions. Thus, the proposed approach represents a viable way to recycle the by-product of the wet phosphoric acid process [33,34]. It is expected that both environmental protection and FAS valorization could be achieved by this approach. 

## 4. Conclusions

In summary, we demonstrated that SiF_4_ hydrolysis at ambient conditions can produce cellular silica, whose walls are composed of nano-sized silica particles. The properties of cellular silica synthesized by using microbubbles as templates, such as cavity size, apparent packing density, and porosity, strongly depend on the nature of the aqueous solutions. The cavity size is negatively proportional to the surface tension; the higher the surface tension, the smaller the size. On the other hand, the thickness of cavity walls seems to be weakly affected by aqueous properties. An attempt was made to introduce aluminum atoms in situ in the second-coordination sphere of Si atoms, but aluminum species occur as alumina instead of Si(*n*Al) species in cellular silica. Cellular silica has an open structure with large pores (hundreds-of-nanometers) that facilitate the transfer of large molecules, including polymers and enzymes, and it could find wide applications in (bio)catalysis, sorption, controlled release, and separations. The presence of aluminum species may endow cellular silica with acid sites; thus, Al-doped cellular silica could be used as an acid catalyst.

## Figures and Tables

**Table 1:** Comparison of various methods to synthesize spherical shell/porous silica.

Method	Raw Materials	Conditions	Ref.
Sodium silicate hydrolysis	Sodium silicate and template	Acid/alkaline environment, template removal	[35]
TEOS hydrolysis	Ethyl orthosilicate and template	Acidic environment, template removal	[36,37]
Stöber method	Base (ammonia, sodium hydroxide), silicon source (TEOS, TMS), and template	Alcohol solution, template removal	[31]
Reverse microemulsion	Surfactant, co-surfactant, oil, water, and template	Microemulsion, template removal	[38]
Selective etching	Silicon source (TEOS, TSD)	Specific pH and temperature	[39,40]
Spray drying	Solvent diluted solution/emulsion	High temperature, dispersion	[41]
Soft-templating	Hexafluorosilicic acid	Ambient conditions	this work

## Data Availability

The data presented in this study are available on request from the corresponding author.

## Supplementary Materials

The following supporting information can be downloaded at: https://www.mdpi.com/article/10.3390/nano12162794/s1, Figure S1: Experimental set-up for synthesis of cellular silica; Figure S2: Drop Images of 0.06 M H_2_SiF_6_ solution and 0.06 M H_2_SiF_6_ with 0.1 M Al(NO_3_)_3_ solution; Figure S3: EDS result of *w*-SiO_2_ and *n*-SiO_2_; Table S1: XRF results of *n*-SiO_2_ sample.
