# Supplementary material for: Synthesis of Cellular Silica Using Microbubbles as Templates
Source: Nanomaterials (Basel). 2022 Aug 15;12(16):2794. doi: 10.3390/nano12162794 (PMC9413465; doi:10.3390/nano12162794)
Supplement: Supplementary file 1 [file nanomaterials-12-02794-s001.zip › nanomaterials-1843858-supplementary.pdf]

Supplementary Materials

Synthesis of Cellular Silica Using Microbubbles as Templates

Zirui Zhao [1], Jiamei Liu [2], Xifeng Xi [1,*], Yulong Wu [3] and Junshe Zhang [1,*]

[1] School of Chemical Engineering and Technology, Xi'an Jiaotong University, Xi'an 710049, China

[2] Instrumental Analysis Center, Xi'an Jiaotong University, Xi'an 710049, China

[3] Institute of Nuclear and New Energy Technology, Tsinghua University, Beijing 100084, China

* Correspondence: xixifeng_xjtu@163.com (X.X.); jzhang08@xjtu.edu.cn (J.Z.)

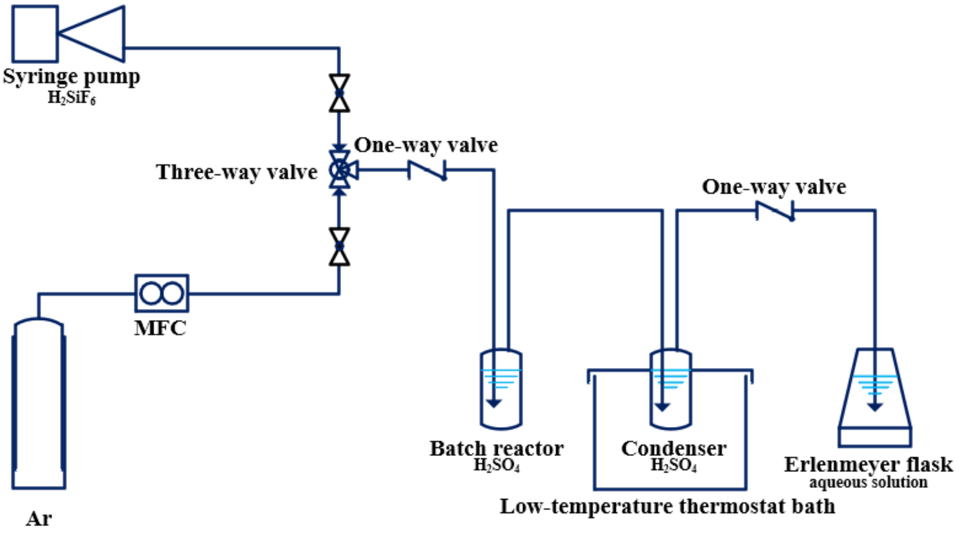

Figure S1. Experimental set-up for synthesis of cellular silica

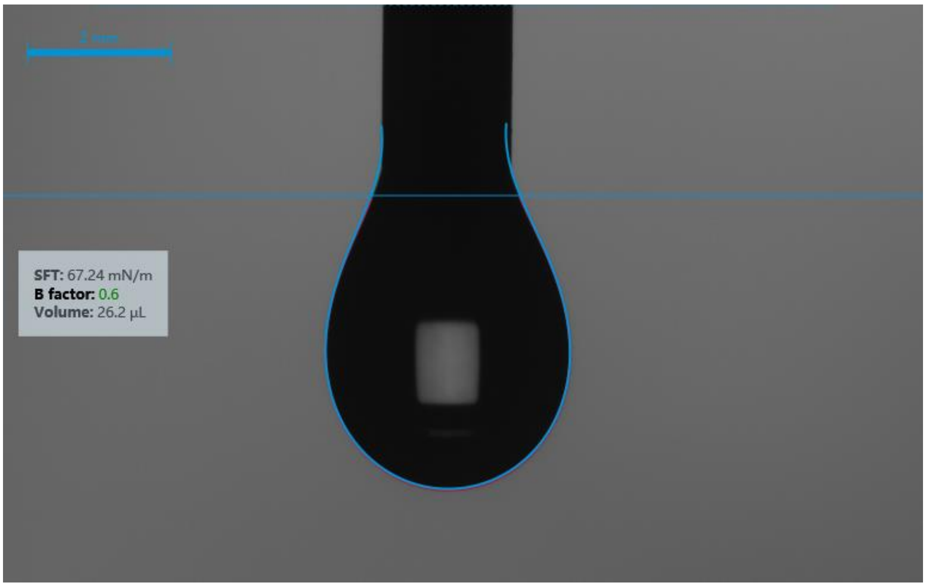

(a)

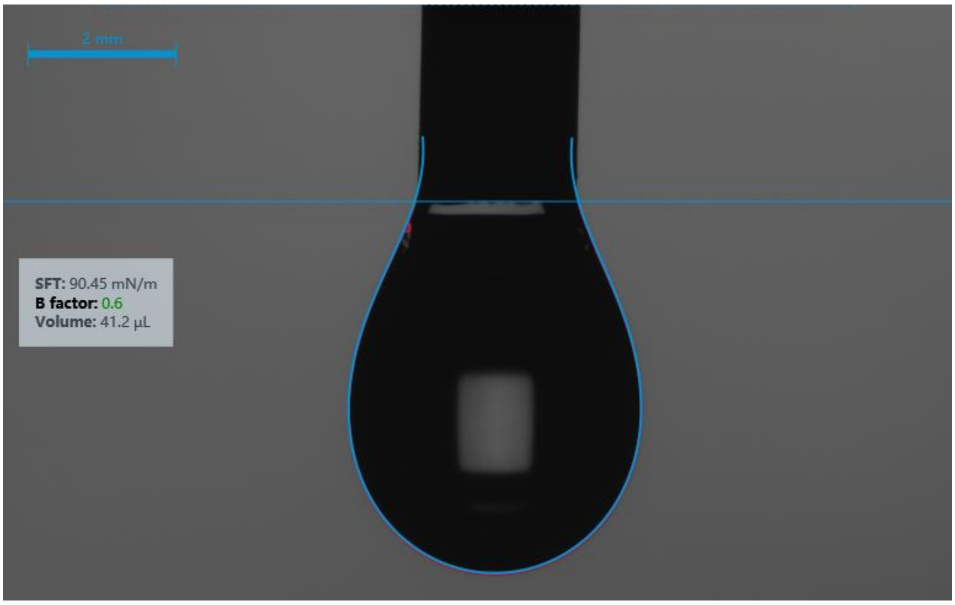

(b)

Figure S2. Drop Images of 0.06 M H_2SiF_6 solution (a) and 0.06 M H_2SiF_6 with 0.1 M $Al(NO_3)_3$ solution (b).

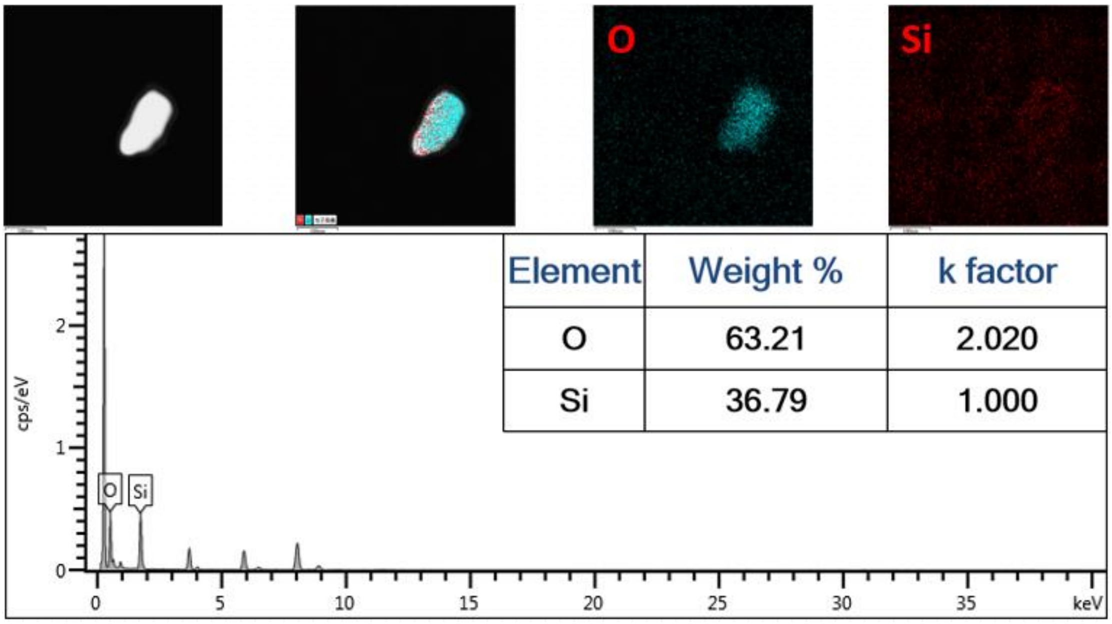

(a)

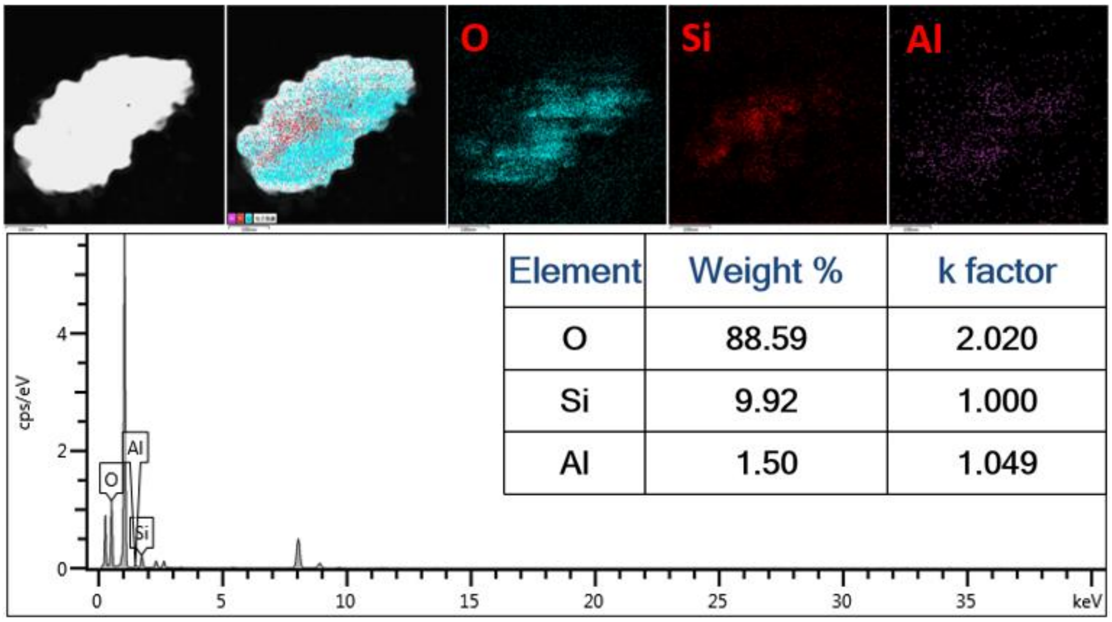

(b)

Figure S3. EDS of (a) *w*-SiO_2 and (b) *n*-SiO_2.

Table S1. Composition of *n*-SiO_2 sample.

Compound	SiO_2	Al_2O_3	P_2O_5	CaO	SO_3	Fe_2O_3	NiO	CuO	ZnO
Mass percentage	92.7	5.71	1.08	0.274	0.2	0.0162	0.004	0.004	0.003
